# Wie Extended-Reality-Anwendungen die Psychotherapie verändern

**DOI:** 10.1007/s00115-025-01924-5

**Published:** 2025-11-26

**Authors:** Simone C. Behrens, Katrin E. Giel, Philipp A. Schroeder, Antonio Capobianco, María Alejandra Quirós-Ramírez, Stephan Streuber, Anne Beck, Bernd Lenz, Thomas Wolbers, André Karger, Steffen Holsteg, Kornelius I. Kammler-Sücker

**Affiliations:** 1 Abt. Psychosomatische Medizin und Psychotherapie, Universitätsklinikum Tübingen, Osianderstr. 5, 72076 Tübingen, Deutschland; 2 Standort Tübingen, Deutsches Zentrum für Psychische Gesundheit (DZPG), Tübingen, Deutschland; 3 Center for Excellence in Eating Disorders Tübingen (KOMET), Tübingen, Deutschland; 4 Fachbereich Psychologie, Universität Tübingen, Tübingen, Deutschland; 5 ICube lab, IGG Team, University of Strasbourg, Strasbourg, Frankreich; 6 Zentrum für Innovative Psychiatrie- und Psychotherapieforschung (ZIPP), Zentralinstitut für Seelische Gesundheit, Mannheim, Deutschland; 7 Fakultät Elektrotechnik und Informatik, Hochschule Coburg, Coburg, Deutschland; 8 Fakultät Gesundheit und Sport, HMU Health and Medical University, Potsdam, Deutschland; 9 Klinik für Abhängiges Verhalten und Suchtmedizin, Zentralinstitut für Seelische Gesundheit, Medizinische Fakultät Mannheim und Universität Heidelberg, Mannheim, Deutschland; 10 Standort Mannheim, Deutsches Zentrum für Psychische Gesundheit (DZPG), Mannheim, Deutschland; 11 Standort Magdeburg, Deutsches Zentrum für Neurodegenerative Erkrankungen (DZNE), Magdeburg, Deutschland; 12 Klinisches Institut für Psychosomatische Medizin und Psychotherapie, Medizinische Fakultät und Universitätsklinikum Düsseldorf, Heinrich-Heine-Universität Düsseldorf, Düsseldorf, Deutschland

**Keywords:** Virtuelle Realität, Psychotherapieforschung, Erweiterte Psychotherapie, Psychologische Behandlung, Digitale mentale Gesundheit, Virtual reality, Psychotherapy research, Enhanced psychotherapy, Psychological treatment, Digital mental health

## Abstract

**Hintergrund:**

Extended Reality (XR) umfasst verschiedene immersive Technologien, die die Wahrnehmung mit virtuellen Elementen erweitern. Die bisherige Forschung hat sich insbesondere auf die mit Virtual Reality unterstützte Expositionstherapie fokussiert, aktuell gibt es jedoch dynamische Entwicklungen sowohl inhaltlicher als auch struktureller Art.

**Methoden:**

Diese narrative Übersichtsarbeit beschreibt von Mitgliedern des Netzwerks „Extended Reality für die Psychotherapie“ genannte inhaltliche und technische sowie strukturelle Entwicklungen in der Erforschung von XR-Anwendungen für die Psychotherapie.

**Ergebnisse:**

Genannt wurden der Einsatz von Mixed-Reality-Technologien und künstlicher Intelligenz und die Entwicklung und Erprobung kreativer therapeutischer Konzepte jenseits des klassischen Expositionsparadigmas. Außerdem werden als strukturelle Entwicklungen die Etablierung standardisierter Prozesse, die Professionalisierung der Entwicklung, Barrieren bei der Implementierung in die Versorgungspraxis und regulatorische Aspekte skizziert.

**Diskussion:**

Die befragten Expert:innen weisen auf technische Weiterentwicklungen und innovative Konzepte hin, die in der aktuellen Psychotherapie so nicht umsetzbar wären. Angesichts der komplexen Technologie, die gleichzeitig multiplen regulatorischen Anforderungen entsprechen muss, bedarf es breit angelegter interdisziplinärer Forschung.

**Zusatzmaterial online:**

Zusätzliche Informationen sind in der Online-Version dieses Artikels (https://doi.org/10.1007/s00115-025-01924-5) enthalten.

## Abstract (en)

**Background:**

Extended reality (XR) encompasses various immersive technologies that enhance perception with virtual elements. Previous research has focused in particular on virtual reality-assisted exposure therapy but there are currently dynamic developments in terms of both content and structure of XR research for psychotherapy.

**Methods:**

This narrative review describes content-related, technical and structural developments in the research of XR applications for psychotherapy, as identified by members of the Extended Reality for Psychotherapy network.

**Results:**

The use of mixed reality technologies and artificial intelligence was mentioned as well as the development and testing of creative therapeutic concepts beyond the classical exposure paradigm. In addition, structural developments, such as the establishment of standardized processes, the professionalization of development, barriers to implementation in healthcare practice and regulatory aspects are outlined.

**Discussion:**

The experts point to technical advances and emerging innovative concepts that would not be feasible in the current psychotherapy. Given the complex technology, which must simultaneously meet multiple regulatory requirements, broad-based interdisciplinary research is needed.

## Hintergrund

Extended Reality (XR) ist ein Überbegriff für verschiedene immersive Technologien, die die Wahrnehmung mit virtuellen Elementen erweitern (siehe Kasten 1). In der Behandlung psychischer Störungen wird XR bisher vor allem in der mit Virtual Reality unterstützten Expositionstherapie eingesetzt. Hierzu gibt es insbesondere im Erwachsenenbereich bereits umfassende Evidenz, der zufolge die virtuellen Szenarien vergleichbar effektiv sind wie die Exposition im echten Leben [1, 6, 36]. Mehrere kommerzielle Anbieter arbeiten inzwischen daran, XR-Anwendungen für die Psychotherapie breit verfügbar zu machen (siehe Tab. 1 für eine Übersicht). Bei insgesamt hoch dynamischem Entwicklungsumfeld wird die Forschungs- und Marktsituation in Deutschland dabei zunehmend komplex und unübersichtlich.

**Tab. 1:** Konferenzen, Netzwerke und kommerzielle Anbieter, die sich mit der Nutzung von XR für die Psychotherapie befassen (Auswahl).

Fachkonferenzen mit XR-spezifischen Symposien und Workshops in Deutschland (2025, Auswahl)	Deutscher Psychosomatik-Kongress (Berlin), TeaP (Frankfurt), Psychologie und Gehirn, Deutscher Psychotherapie-Kongress (Berlin), DGPPN-Kongress (Berlin), DAG/DGESS-Kongress (Stuttgart)
XR-spezifische Konferenzen (2025, Auswahl)	ShiftMedical (Heidelberg), MindXR (Berlin), XR Expo (Stuttgart), Virtual Reality in Mental Health (Helsingborg), VR Summit (Bochum), Wuertual Reality XR-Meeting (Würzburg)
Interdisziplinäre Netzwerke	Arbeitsgruppe „Extended Reality in der Psychotherapieforschung“, Extended Reality Berlin-Brandenburg e. V., Kompetenzzentrum für virtuelle Realität und Kooperatives Engineering w.V. (VDC)
Kommerzielle Anbieter von XR für die Psychotherapie	Invirtu, Lab E GmbH, VR Coach, neomento, PsyLaris, PsyCurio, Magic Horizons, VTplus

Ziel dieser narrativen Übersichtsarbeit ist es, aktuelle inhaltliche und strukturelle Entwicklungen in der Forschung zum Einsatz von XR-Technologien in der Psychotherapie darzustellen und die Bedeutung koordinierter interdisziplinärer Forschung in diesem Bereich herauszuarbeiten. Dafür wurden über das interdisziplinäre Netzwerk „Extended Reality in der Psychotherapieforschung“ Expert:innen eingeladen, aktuelle Entwicklungen in der Anwendung von XR für die Psychotherapieforschung beizutragen. Die zunächst schriftlichen Beiträge wurden in mehreren offenen mündlichen Diskussionsrunden strukturiert und schließlich in diesem Beitrag verschriftlicht.

## Das Netzwerk „Extended Reality in der Psychotherapieforschung“

Das Netzwerk „Extended Reality für die Psychotherapie“ hat sich aus der 2021 im Rahmen der Deutschen Gesellschaft für Essstörungsforschung (DGESS) gegründeten Arbeitsgruppe „Virtuelle Realität in der Essstörungsforschung“ entwickelt. Kernanliegen des interdisziplinär angelegten Netzwerks ist die Förderung der Nutzung von XR in der Psychotherapieforschung. Mitglieder des Netzwerks wurden im Peer-to-peer-Verfahren rekrutiert und kommen aus akademischen Institutionen bundesweit sowie aus angrenzenden europäischen Staaten. Aktuell nehmen mehr als 10 Arbeitsgruppen regelmäßig an den Netzwerkaktivitäten teil. Die Mitglieder sind außerdem im Deutschen Zentrum für Psychische Gesundheit (DZPG) sowie in verschiedenen thematisch assoziierten Fachgesellschaften engagiert, neben der DGESS etwa in der DGPs, der DGPPN, dem DKPM, der DAG, der DG Sucht, der DGVT, der EPA, der DKH (AG Psychoonkologie), sodass deren einschlägige Aktivitäten querschnittlich harmonisiert werden können. Projekte der Mitglieder umfassen unter anderem die Erforschung und Entwicklung innovativer XR-Anwendungen, theoretische Grundlagen und praktische Standards für die klinische Forschung mit XR sowie die Nutzung von XR für die Erforschung und Behandlung von Essstörungen, von Suchterkrankungen, Depressionen, Persönlichkeitsstörungen, chronischen Schmerzsyndromen sowie im Rahmen psychoonkologischer Betreuung.

## Technische Entwicklung: Mixed Reality und künstliche Intelligenz verändern XR-Anwendungen

Bisher nutzen die meisten XR-Anwendungen für Psychotherapie Virtual Reality, d. h. die Nutzer:innen werden immersiv in eine vollständig computergenerierte virtuelle Umgebung versetzt, um dort bestimmte Erfahrungen zu machen oder Übungen durchzuführen. Im Gegensatz dazu ermöglichen Mixed Reality und Augmented Reality den Nutzer:innen, mit virtuellen Agenten, Objekten oder Szenarien zu interagieren, während sie gleichzeitig mit ihrer realen Umgebung verbunden bleiben. Dies kann zu natürlicheren affektiven und Verhaltensreaktionen führen [12]. Diese Integration bietet Potenzial in allen therapeutischen Übungsszenarien, in denen Immersion, Interaktivität und ökologische Validität entscheidend für die Wirksamkeit sind.

Um XR-Anwendungen natürlicher und intuitiver zu gestalten, wird zunehmend künstliche Intelligenz eingesetzt. Anwendungsbereiche künstlicher Intelligenz sind etwa die Integration von Biofeedback [20], die Echtzeitauswertung mimischer Reaktionen [15, 33], die Generierung und Echtzeitanimation biometrisch akkurater virtueller Menschen (Avatare; [25, 27]), der Einsatz von Large-Language-Models für soziale Interaktionen [31] oder die Sprachnavigation [13] sowie Übungen für die Emotionsregulation in natürlich wirkenden Interaktionen [29]. Auch Avatare können durch den Einsatz künstlicher Intelligenz natürlicher und interaktiver wirken [4, 7]. Anwendungsbeispiele für diese Entwicklungen sind im Infokasten 2 dargestellt.

## Inhaltliche Entwicklung: Optimierung etablierter Therapietechniken und Entwicklung innovativer Innovationen

Nachdem für die virtuelle Expositionstherapie nachgewiesen werden konnte, dass der therapeutische Nutzen der konventionellen Exposition ebenbürtig ist [6], wird aktuell daran gearbeitet, die spezifischen Möglichkeiten der Technik für die Optimierung etablierter Therapietechniken einzusetzen. In der virtuellen Expositionstherapie erleben die Patient:innen wie in der konventionellen Exposition virtuelle Umgebungen mit variablem Schwierigkeitsgrad, in denen angstauslösende Situationen wie etwa Menschenmengen, Höhen oder bestimmte Tiere vorkommen. Die Behandler:innen begleiten die Patient:innen dabei, ihre Emotionen funktional und insbesondere ohne Vermeidungsverhalten zu regulieren.

Optimierende Entwicklungen zielen nun darauf ab, den Patient:innen und Therapeut:innen durch Interaktionen mit virtuellen Coaches oder Chatbots mehr eigene Gestaltungsmöglichkeiten zu geben. Die Nutzer:innen können dann individuelle Anpassungen der Szene oder des Schwierigkeitsgrades selbst vornehmen, z. B. das Gegenüber einen individuell schwierigen Satz aussprechen lassen oder ein gefürchtetes Tier näher kommen lassen. Außerdem werden konzeptuelle Erweiterungen getestet, etwa durch die Integration von Biofeedback und die Vergabe von Belohnungen für gemeisterte Aufgaben oder Level *(Gamification;* [10]).

Diese Entwicklung erstreckt sich auch auf die Adaptation anderer etablierter Therapietechniken: Im Rahmen der *psychodynamischen Psychotherapie* können virtuelle soziale Situationen eingesetzt werden, etwa um ein realitätsnahes Erleben interpersoneller Beziehungsthemen zu erreichen. Erweiternd zur konventionellen Umsetzung sind dabei Pausen, Perspektivwechsel und das wiederholte Ausprobieren alternativer Reaktionen möglich. Diese Komponenten sollen gemeinsam Einsicht in eigene Beziehungsmuster und eine bewusste Gestaltung von Beziehungen fördern [14].

Auch die *Cue Exposure *adaptiert eine analog bereits etablierte Therapietechnik in XR, womit die Technologie auch für Betroffene von Zwangserkrankungen oder Suchterkrankungen erschlossen wird [2, 8, 24, 32]. Die XR-Technologie wird dabei genutzt, um schwierige Situationen zu simulieren und deren Bewältigung zu üben. Die Patient:innen sollen dabei „Not-just-right“-Gefühle, Craving oder den Drang nach gewichtsregulierendem Verhalten erleben und funktionale Reaktionen darauf einüben. Der Einsatz von XR soll die praktische Durchführbarkeit der Cue Exposure erheblich verbessern. Allerdings erfordert die Induktion komplexer, situationsabhängiger Empfindungen und Kognitionen eine aufwendige Entwicklung mit Phase-I- und Phase-II-Studien.

*Handlungsbasierte XR* verfolgt den Ansatz, automatisierte Routinen durch Übung und Training zu verändern und dabei in ökologisch valide Szenarien zu übersetzen. Approach-Avoidance-Trainings, Rollenspiele und Szenen zur Förderung der Perspektivübernahme im Rahmen systemisch motivierter Aufstellungen oder gezielt eingesetzter Erste-Person-Avatare können neue Handlungsweisen und Interaktionsmuster schulen oder eine verbesserte Selbstwahrnehmung und Selbstakzeptanz induzieren [19]. Dieser Ansatz nutzt häufig bestehende Grundlagenforschung und experimentelle Therapiemodule, die durch die Adaptation in XR praktisch für die Nutzung im Rahmen einer Psychotherapie erschlossen werden.

Die XR-Technologie ermöglicht es, virtuell veränderte Körper aus der Ich- oder Spiegelperspektive zu erleben. Dieses sog. *Embodiment* wird zur Erforschung und ggf. therapeutischen Manipulation interner Körperrepräsentationen und des Selbstbilds eingesetzt. So können etwa individualisierte Avatare mit individuellem Zielgewicht genutzt werden, um Behandlungsziele und dafür nötige Schritte bei Ess- und Gewichtsstörungen emotional erfahrbar zu machen [4, 11, 18]. Bei chronischen Schmerzen werden virtuelle Doppelgänger genutzt, um komplexe Interaktionen von Körperwahrnehmung und körperbezogenen Überzeugungen mit weiteren biopsychosozialen Faktoren therapeutisch zu beeinflussen [16, 23, 30].

Der massiv aufmerksamkeitslenkende Charakter insbesondere virtueller Realität wird zunehmend für die *Förderung der Emotionsregulation und Entspannung* eingesetzt. Zum Beispiel wurden entspannende Szenarien bei einer Vielzahl psychischer Störungen mit ähnlichen oder größeren Effekten wie analoge Entspannung eingesetzt [28]. Es gibt aber auch erste Arbeiten dazu, Erlebensübungen aus der Akzeptanz-Commitment-Therapie (ACT) als Mini-Games in virtueller Realität zur spielerischen Selbstdistanzierung von belastenden Gedankenzirkeln einzusetzen oder Entspannungsübungen gezielt zur Emotionsregulation einzusetzen [21, 26].

## Strukturelle Entwicklungen: Standardisierung, Professionalisierung und regulatorische Entwicklungen

Frühe XR-Studien berichteten meist Effekte einer XR-gestützten Intervention in einer kleinen Stichprobe. Inzwischen hat sich für die Entwicklung von XR-Anwendungen ein gestuftes Vorgehen etabliert, das an die Entwicklung von Medizinprodukten angelehnt ist. Dabei wird ein besonderer Fokus auf nutzendenzentriertes Design gelegt [3, 5] und es wurden kürzlich auch operative Standards für das Berichten von XR-Entwicklungen entwickelt [34]. Diese Richtlinien tragen der komplexen Natur von XR-Anwendungen Rechnung und bieten eine Anerkennung der kleinschrittigen und oft langwierigen Entwicklung. Die konsequente Anwendung soll dazu beitragen, wesentliche Wirkfaktoren der XR-Anwendungen zu identifizieren die zugehörigen Studienergebnisse replizierbar zu machen.

Insgesamt berichten die Expert:innen eine Professionalisierung der translationalen Entwicklung von XR-Anwendungen, die sich nicht zuletzt in einer Zunahme kommerzieller Anbieter und themenspezifischer Fachkonferenzen widerspiegelt (Tab. 1). Obwohl eine Vielzahl der Behandler XR-Technologien positiv gegenübersteht, werden in Umfragen eine Vielzahl strukturell-organisatorischer Hürden für die Implementierung in der Versorgung genannt: individuelle Barrieren (Wissen, Training, Zeit, persönliche Gründe), finanzielle Aspekte (Kosten, Kosten-Nutzen-Verhältnis, Erstattungsfähigkeit), therapeutische Aspekte (klinische Evidenz, Berücksichtigung in Leitlinien, Passung ins Therapiekonzept, Sorge um therapeutische Beziehung) und technologische Barrieren (Stabilität und Nutzerfreundlichkeit der Systeme, Nebenwirkungen; [9, 17, 35]). Vielen dieser Barrieren könnte mit strukturierter, vernetzter Forschung i. S. eines präzisionsmedizinischen Ansatzes [37] effizient begegnet werden, was die Bedeutung der einschlägigen interdisziplinären Netzwerke unterstreicht.

Eine große Herausforderung der Erforschung von XR-Anwendungen für die Psychotherapie über die unmittelbare Entwicklung und Pilotierung hinaus sind regulatorische Hürden. Abhängig vom Gestaltungsgrad der Behandler:innen fallen XR-Umgebungen unter die EU-Vorgaben zu Medizinprodukten. Die damit einhergehenden Berichts- und Dokumentationspflichten und die Anforderungen einer dynamischen Softwareentwicklung erzeugen hohe Aufwände. In Deutschland, aber auch in den europäischen Nachbarländern, existieren verschiedene öffentlich geförderte Vernetzungsinitiativen und spezifische Förderformate für Kooperationsprojekte zwischen kleinen und mittleren Unternehmen, wie etwa das CyberValley in Deutschland oder Biovalley in Frankreich. Die translationale Entwicklung und Erforschung digitaler Gesundheitsanwendungen und erweiterter Psychotherapie sind außerdem Schwerpunkte des neu gegründeten DZPG.

## Fazit für die Praxis

Extended Reality hat sich in Teilbereichen der Psychotherapie bereits empirisch bewährt und hat das Potenzial, mit innovativen Therapiekonzepten spürbare Verbesserungen bestehender Therapiekonzepte zu generieren. Die befragten Expert:innen weisen hierbei auf technische Weiterentwicklungen hin, aber auch auf innovative Konzepte, die in der aktuellen Psychotherapie so nicht umsetzbar wären. Angesichts der komplexen Technologie, die gleichzeitig multiplen regulatorischen Anforderungen entsprechen muss, bietet sich ein gestuftes Vorgehen gemäß konsentierter Prozesse an. Dabei bedarf es eines großen interdisziplinären Netzwerks an Forschenden aus klinischen und technischen Disziplinen sowie verschiedener Kooperationsprojekte mit der Industrie. Initiativen wie das Netzwerk „Extended Reality für die Psychotherapie“ können diesen Prozess unterstützen.

## Supplementary Information

Infokasten 1: Begriffsdefinitionen. Infokasten 2: Anwendungsbeispiele für technische und inhaltliche Entwicklungen
