# Supplementary material for: How extended reality applications make a difference in psychotherapy
Source: Nervenarzt. 2025 Nov 26;97(6):585–9. [Article in German] doi: 10.1007/s00115-025-01924-5 (PMC13719835; doi:10.1007/s00115-025-01924-5)
Supplement: Supplementary file 1 [file 115_2025_1924_MOESM1_ESM.docx]

**Kasten 1: Begriffsdefinitionen**

***„Extended Reality“ (XR)*** ist ein Überbegriff für verschiedene Technologien zur Erweiterung der Wahrnehmung, die zum Beispiel zusätzliche digitale Informationen (Augmented Reality) oder Objekte (Mixed Reality) einblenden oder auch komplett immersive virtuelle Umgebungen (Virtual Reality) zeigen. Meist erfolgt dies über visuelle Darbietung, zusätzlich können auditive, olfaktorische oder taktile Stimuli integriert werden [21].

***„Augmented Reality”*** beschreibt ergänzende Einblendungen in das Sichtfeld der Benutzer:innen etwa von zusätzlichen virtuellen Objekten oder Hinweisen. Technische Umsetzungen sind mit dem Smartphone oder auch speziellen Augmented Reality Brillen möglich.

***„Mixed Reality”*** verwendet ergänzende interaktive Objekte in der physischen Welt: Ein virtueller Hund unter dem Tisch würde also nur eingeblendet, wenn man auch unter den Tisch schaut und könnte sich auch auf den Nutzer:innen zu- oder wegbewegen oder auf seine Handlungen reagieren. Häufig werden leistungsstarke Smartphones eingesetzt, aber auch spezielle Brillen, sogenannte Head-Mounted Displays (“VR-Brillen“) kommen technisch infrage.

***„Virtual Reality”*** zeigt Nutzern eine vollständig computer-generierte Realität, entweder als 360°-Video oder als komplexe 3D-Welt Umgebung ähnlich wie in einem Computerspiel. Die Darbietung erfolgt manchmal in speziell ausgerüsteten Räumen („Cave“), oder alternativ über Head-Mounted Displays („VR-Brillen“).

***Kasten 2: Anwendungsbeispiele für technische und inhaltliche Entwicklungen***

- ***Expositionstherapie in Mixed Reality und Augmented Reality:*** Mixed Reality und Augmented Reality ermöglichen die kontrollierte Exposition mit virtuellen Reizen im aktuellen, realen Umfeld des Nutzers. Dies soll die Generalisierung der erlernten Bewältigungsstrategien gegenüber virtueller Realität, die eine vollständige Immersion ermöglicht und damit von der alltäglichen Umgebung abgespalten ist, verbessern. Die Flexibilität der augmentierten Umgebung ermöglicht zudem schrittweise Anpassungen an den Schwierigkeitsgrad oder die Intensität der Exposition.
- ***künstliche Intelligenz für soziale Szenarien:*** Mit interaktiven, durch künstliche Intelligenz natürlich agierenden virtuellen Menschen können Patient:innen ihren Umgang mit komplexen sozialen Interaktionen üben, wie z. B. den Umgang mit Zurückweisung, den Aufbau von Beziehungen oder die Lösung von Konflikten. Virtuelle Menschen, die mit realistischen verbalen und nonverbalen Verhaltensweisen programmiert sind, können Patient:innen in diesen Szenarien herausfordern, während gleichzeitig Sicherheit und Kontrolle gewährleistet werden.
- ***Embodiment von gesundem Gewicht für die Therapie von Ess- und Gewichtsstörungen:*** Personen mit Ess- und Gewichtsstörungen müssen eine Vielzahl von Änderungen in ihrem Ernährungs- und Bewegungsverhalten dauerhaft umsetzen, um ein gesundes Gewicht zu erreichen. Dabei sind die mittelfristigen positiven Auswirkungen für die Betroffenen oft gefühlt „weit weg“. Mit individualisierten, animierten Avataren, die aus der Ich- und Spiegelperspektive erlebt werden, kann die Gewichtsentwicklung visuell vorweggenommen werden. Je nach individuellen Bedarfen wird dies genutzt, um negative Erwartungen zu entkräften oder um die Motivation und Selbstwirksamkeitserwartung für konkrete Schritte zu stärken.
- ***Rollenspiele für die Arbeit an Beziehungskonfliktthemen.*** Interaktive, soziale Situationen, die von der Therapeut:in gesteuert werden, sollen im Rahmen der psychodynamischen Depressionstherapie ein realitätsnahes Erleben des eigenen Beziehungsthemas ermöglichen. Die virtuellen Gesprächspartner:innen können individuell angepasst werden. Eine Perspektivwechsel-Funktion unterstützt dabei die therapeutische Nachbesprechung der Rollenspiel-Situation. Die Auswertung von Sprachdaten der Patient:innen hinsichtlich der Stimmung und Depressivität wird den Therapeut:innen anschließend in einem Reporting-Modul zur Verfügung gestellt.
